# Compliance with Australian Orthopaedic Association guidelines does not reduce the risk of venous thromboembolism after total hip and knee arthroplasty

**DOI:** 10.1038/s41598-024-54916-x

**Published:** 2024-03-12

**Authors:** Helen Badge, Tim Churches, Justine M. Naylor, Wei Xuan, Elizabeth Armstrong, Leeanne Gray, John Fletcher, Iain Gosbell, Chung-Wei Christine Lin, Ian A. Harris

**Affiliations:** 1 Whitlam Orthopaedic Research Centre, 1 Campbell Street, Liverpool, 2071 Australia; 2 South Western Sydney Clinical School, UNSW, 1 Elizabeth Street, Liverpool, 2071 Australia; 3 Ingham Institute for Applied Medical Research, 1 Campbell Street, Liverpool, 2071 Australia; 4 Australian Catholic University, 8-20 Napier Street, North Sydney, 2060 Australia; 5 School of Public Health, The University of New South Wales, UNSW Kensington Campus, 2033, Botany Street, Kensington, NSW 2052 Australia; 6 South Western Sydney Local Health District, 1 Elizabeth Street, Liverpool, 2071 Australia; 7 University of Sydney, Fisher Road, Camperdown, NSW 2006 Australia; 8 Westmead Hospital, Cnr Hawkesbury Road and Darcy Road, Westmead, NSW 2145 Australia; 9 Western Sydney University, Campbelltown, NSW 2560 Australia; 10 Sydney School of Population Health, The University of Sydney, Edward Ford Building (A27) Fisher Road, Camperdown, NSW 2006 Australia

**Keywords:** Primary total hip arthroplasty, Primary total knee arthroplasty, Clinical guidelines, Venous thromboembolism, Prophylaxis, Surgical complications, Health services, Osteoarthritis

## Abstract

Preventing avoidable venous-thrombo-embolism (VTE) is a priority to improve patient and service outcomes after total hip and total knee arthroplasty (THA, TKA), but compliance with relevant clinical guidelines varies. This study aims to determine the degree to which prophylaxis was compliant with Australian Orthopaedic Association (AOA) VTE prophylaxis guidelines and whether non-compliance is associated with increased risk of VTE. A prospective multi-centre cohort study of adults with osteoarthritis undergoing primary TKA/THA was completed at 19 high-volume public and private hospitals. Data were collected prior to surgery and for one-year post-surgery. Logistic regression was undertaken to explore associations between non-compliance with AOA VTE prophylaxis guidelines and symptomatic 90-day VTE outcomes. Data were analysed for 1838 participants from 19 sites. The rate of non-compliance with all clinical guideline recommendations was 20.1% (N = 369), with 14.1% (N = 259) non-compliance for risk-stratified prophylaxis, 35.8% (N = 658) for duration, and 67.8% (N = 1246) for other general recommendations. Symptomatic VTE was experienced up to 90-days post-surgery by 48 people (2.6%). Overall guideline non-compliance (AOR = 0.93, 95%CI = 0.4 to 1.3, *p* = 0.86) was not associated with a lower risk of symptomatic 90-day VTE. Results were consistent when people with high bleeding risk were excluded (AOR = 0.94, 95%CI = 0.44 to 2.34, *p* = 0.89). Non-compliance with the AOA VTE prophylaxis guidelines was not associated with risk of 90-day VTE after arthroplasty. This counterintuitive finding is concerning and necessitates a rigorous review of the AOA VTE prevention clinical guideline.

## Introduction

Venous thromboembolism (VTE) (including deep vein thrombosis [DVT] and pulmonary embolism [PE]) remains one of the major causes of morbidity, mortality, and costs in total hip and knee arthroplasty (THA/TKA)^1^. Compliance with clinical practice guidelines (CPG) has been inconsistent due to concerns with the quality and recency of evidence, the relevance of symptomatic versus asymptomatic VTE, and the need to balance the risks and benefits of VTE prophylaxis against the risk of bleeding and surgical site infection^2–4^.

The Australian Orthopaedic Association (AOA) have endorsed the Arthroplasty Association of Australia guidelines for VTE prophylaxis for THA/TKA^5^. Many surgeons use the AOA VTE guidelines^6^, so it is important to investigate the level of compliance with these guidelines, and whether non-compliance is associated with VTE. Our study group has previously shown that non-compliance with the (then current) National Health and Medical Research Council (NHMRC) VTE prevention clinical guidelines^7^ was associated with higher rates of VTE after THA/TKA. The NHMRC VTE clinical guideline is a high quality guideline developed using rigorous methods^8,9^. The quality of the AOA guideline cannot be determined as there is no published information on the methods used or quality evidence underpinning the recommendations. The key difference between the two guidelines is that the NHMRC guidelines recommended only potent anticoagulants as all THA and TKA recipients are considered at high risk of VTE, whereas the AOA guidelines provides criteria to stratify patient risk, and recommends either potent anticoagulants or aspirin (an antiplatelet agent) with a sequential compression device for people assessed as being at routine VTE risk^5,7^. Using the same prospective cohort, this study aims to determine the magnitude of non-compliance with AOA VTE prophylaxis CPG and determine if there is an association between non-compliance and VTE after THA/TKA^5^.

## Methods

### Registration and data collection

A prospective observational cohort study of people undergoing elective primary THA/TKA for osteoarthritis in one of 19 high-volume institutions in Australia was performed to examine the relationship between non-compliance with several VTE prophylaxis and antibiotic CPG, and patient outcomes. In this paper we explored the association with the AOA VTE CPG and did not analyse any antibiotic data. Eligible sites included private and public Australian hospitals with high annual surgical volume (> 275 cases) of THA/TKA. Participant inclusion criteria were adults (≥ 18 years) with a primary diagnosis of osteoarthritis, undergoing primary THA/TKA; sufficient English to comprehend the protocol; and available to participate in 12 month follow-up.

Data were collected prospectively prior to surgery, during the acute admission and via telephone follow at 35, 90, and 365 days post-surgery. The accuracy of patient-reported and acute complications was verified by medical record audits at all sites and by contacting surgeons, primary care, and hospitals. The study protocol was registered prior to commencement^10^ and ethical approval was obtained from nine relevant human research ethics committees. The study has been described in accordance with strengthening the reporting of observational studies in epidemiology guidelines^11^.

### Criteria for primary outcome and compliance with clinical guidelines

The primary outcome was any symptomatic VTE event (PE/DVT) up to 90 days post-surgery. The exposure was non-compliance with the risk-stratified recommendations of the AOA VTE clinical guidelines for THA/TKA^5^ (Table 1). Using criteria identified in the AOA VTE clinical guidelines the level of risk was categorised for each participant as routine or high for VTE, or high for bleeding. There were three dichotomous elements of compliance (Table 1): patient-level risk-stratified compliant prophylaxis, compliant duration of prophylaxis and compliance with other general recommendations (Table 1)^5^. For people who did not have a VTE or had a VTE after the recommended period of prophylaxis, the duration of prophylaxis was considered compliant if they received at least one prophylactic drug for at least 21 days from the day of surgery. For people who had a VTE during the recommended period of prophylaxis, the duration was calculated up until the day of VTE diagnosis, as further use of an anticoagulant was considered treatment rather than prophylaxis. If they started VTE treatment the same day or day after prophylaxis ceased, they were considered compliant for duration. The AOA recommended duration was three to six weeks, however we allowed a minimum of three weeks as compliant, without penalising for longer term use. Overall guideline compliance required the person to be compliant with all three elements. The primary predictor was the dichotomous overall AOA VTE clinical guidelines compliance variable.

**Table 1:** Criteria for determining risk and compliance with Arthroplasty Association of Australia (AOA) Prevention of VTE Guideline recommendations (2018).

Criteria for determining patient risk	
High risk of PE	Does not meet criteria for high bleeding risk, and
	One or more major criteria: hypercoagulability conditions, premorbid haemorrhagic stroke, congestive obstructive pulmonary disease (COPD), or
	Three or more minor criteria: History VTE, tamoxifen/oestrogen therapy, body mass index (BMI) ≥ 30, A
High risk of bleeding	Known bleeding disorder
Routine VTE risk	Does not meet criteria for high VTE/bleeding risk
Criteria for AOA recommended prophylaxis (prophylaxis)	
Routine VTE (AOA 2)	Sequential compression device (SCD)
	Aspirin 100–300 mg/day OR
	Potent anticoagulant [low molecular weight heparin (LMWH), warfarin, direct oral anticoagulant (DOAC)]
High VTE risk (AOA 3)	SCD in combination with warfarin, LMWH, DOAC
High bleeding risk (AOA 4)	SCD only
Criteria for patient-level risk-stratified compliant drug	
(i) Patients considered routine VTE risk who received routine VTE risk compliant prophylaxis	
(ii) Patients considered high VTE risk who received high VTE risk compliant prophylaxis	
(iii) Patients considered high bleeding risk who received high bleeding risk compliant VTE prophylaxis	
Received recommended prophylaxis^‡^	Proportion of all participants who received risk-stratified compliant prophylaxis (as per i., ii., iii. above)
Compliance with general recommendations^‡^	
Recommended duration^‡^	Duration VTE prophylaxis 3–6 weeks, compliance allowed a minimum of 21 days or ceased VTE prophylaxis to commence treatment for VTE
General recommendations^‡^	Early mobilisation (day 0 or 1)
	Spinal anaesthesia (or non-use considered a patient-appropriate variation people taking warfarin)
	Bleeding mitigation (use of tranexamic acid (TXA))
	If taking, ceased clopidogrel by 7 days and warfarin by 5 days prior to surgery

^‡^Three primary predictor compliance variables.

Anaesthesia was dichotomised into neuraxial anaesthesia received or not from nine combinations of general anaesthetic (GA), spinal, epidural, regional nerve block and sedation. Compliance with anaesthetic recommendations included people who received neuraxial anaesthesia or were taking warfarin preoperatively, where use of neuraxial anaesthesia may have been considered contraindicated. Some elements of the recommendations were excluded if they were not relevant for this study or if the data was unavailable, such as current metastatic cancer and individual normalised ratios.

Computer-based algorithms were developed to automatically generate compliance results to ensure the consistent application of the criteria for risk assessment and guideline compliance^12^ (10.26190/mddw-by48). The a priori sample size calculations for the primary study were used for the purpose of this secondary data analysis^10^.

### Data analyses

All data were entered into a designated REDCap database^13^. All analyses were conducted using the R Environment for Statistical Computing (version 3.6.1)^12^. Descriptive statistics were calculated to profile site-level and participant-level characteristics. Results were presented as median and inter-quartile values. Some variables (bilateral joint, smoking status, American Society of Anesthesiology score [ASA], education) were collapsed to allow for adequate group size or clinically meaningful groups. The dataset was reduced to include only those for whom risk-stratified compliance could be determined.

Bivariable (unadjusted) analyses were performed including chi-squared tests or t-tests, using Fisher’s exact test. We conducted multivariable logistic regression to explore associations between overall AOA VTE CPG non-compliance and the risk of VTE outcomes. The primary predictor and other factors identified on bivariable (unadjusted) analysis with a *p*-value < 0.25 were entered into a backwards, stepwise multivariable logistic regression model (using the Akaike information criterion–AIC) forcing only the main predictor into the model (Supplementary Table 4).

The rate of missing data was low (1%) and appeared to be random. Missing data were imputed using multiple imputation by chained equations and model selection was performed using one of the imputed datasets. Effect estimates were pooled estimates from the five imputed datasets. Using the final model, interaction terms for the primary non-compliance predictor against each other variable were tested for each analysis. Sensitivity analyses were performed using THA and TKA separately, excluding participants with premorbid history of VTE, and excluding participants who received a routine doppler ultrasound (DUS). Further sensitivity analyses were performed using complete case analysis and using Bayesian information criterion (BIC) for model selection. A final sensitivity analysis included using the three elements of compliance (prophylaxis, duration, general recommendations) as separate predictors, instead of the overall AOA VTE compliance variable. A deidentified version of the data set and the full R code for all analyses are available (10.26190/mddw-by48).

### Ethics approvals and consent to participate

Ethical approval was obtained from nine ethics committees. Austin Health Human Research Ethics Committee (EC00204)—Approval LNR/14/Austin/208; Barwon Health Human Research Ethics Committee (EC00208)—Approval LR13/82; Calvary Health Care Adelaide Human Research Ethics Committee (EC00302)—Approval 13_CHREC-E007; Calvary Health Care Tasmania Clinical and Research Ethics Committee—Approval 2:05:13 and 5:13:12; Epworth HealthCare Human Research Ethics Committee (EC00217) Approval LR138- 13; Hunter New England Human Research Ethics Committee (EC00403) Approval LNR/HNE/390; St Vincent's Health and Aged Care HREC (EC00324) Approval HREC #13/10; Sydney Adventist Hospital Group Human Research Ethics Committee (EC00141)—Approval 2013-016; The Prince Charles Hospital HREC (EC00168) Approval HREC 13-141; Prior to data collection informed written consent was obtained from eligible participants and the signed consent form was witnessed by the site coordinator.

## Results

### Sample ascertainment

**Figure 1:**
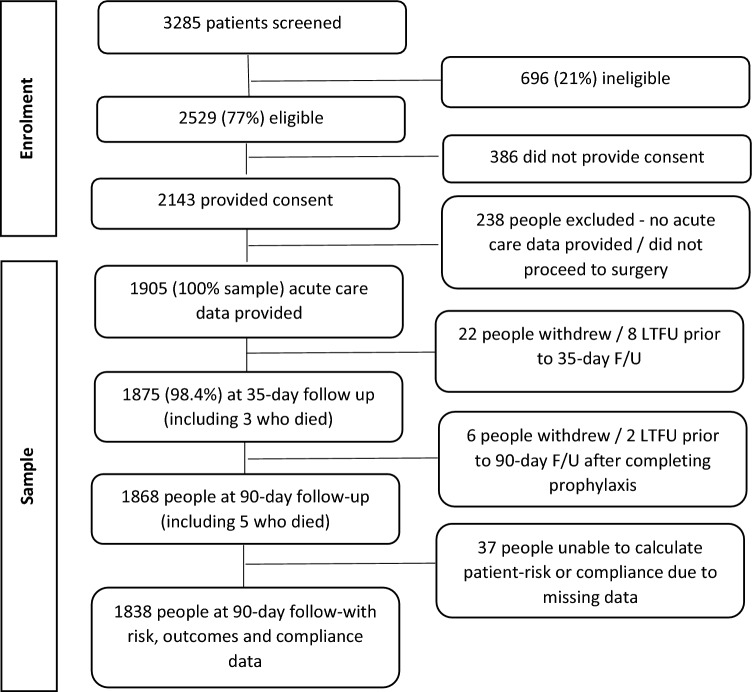
Participant recruitment, eligibility, and participation.

In total, 3285 patients were screened and 2529 (77%) were eligible. Of the 2143 who provided consent, acute care data were received for 1905 participants. Participants were excluded if they did not proceed to surgery or no acute-care data were received (238 participants), they did not have any post-acute follow up (30 people, 1.6%) so 1875 participants were included in the sample. A further 38 participants (2%) were excluded as compliance could not be calculated. Data were analysed for 1837 (97.9%) consenting participants (Fig. 1). Missing data was less than 2.2% for all variables.

### Sites, surgeon and participant characteristics

Site, surgeon, and participant characteristics are provided in Table 2.

**Table 2:** Participant characteristics.

Characteristics	Description	N (%) or median (IQ)
Age (years)		67.7 (61.0, 74.0)
Sex	Female	992 (54.0%)
Insurance	Public	844 (44.9%)
	Private health	993 (54.1%)
Education (N = 1830)	No post-school	880 (48.1%)
	Post school	949 (51.9%)
BMI		30 (26, 34)
Current smoker (N = 1828)	No	1676 (91.7%)
	Yes	152 (8.3%)
Comorbid Conditions	Cardiac	460 (25.0%)
	Heart failure	12 (0.7%)
	History stroke	111 (6.0%)
	Hypercoagulability conditions: (N = 1818)	18 (1%)
	von Willebrand Disease	1
	Factor V deficiency	2 (0.1%)
	Platelet dysfunction or low count	8 (0.4%)
	Other bleeding disorder (not specified)	9 (0.5%)
	Previous VTE (N = 1836)	146 (8.0%)
	Diabetes	301 (16.4%)
	Hypertension	1115 (60.7%)
	Hypercholesterolaemia	689 (37.5%)
	Renal	59 (3.2%)
	Liver	48 (2.6%)
	Any cancer (current)	40 (2.2%)
	Any cancer (history) (N = 1836)	216 (11.8%)
	Respiratory	337 (18.3%)
	COPD/emphysema	65 (3.5%)
	Anxiety/depression	344 (18.7%)
	Other mental health disorder	22 (1.2%)
	Gastro-intestinal reflux disorder	476 (25.9%)
	Sleep apnoea	127 (6.9%)
	Neurological	53 (2.8%)
	Musculoskeletal (N = 1873)	881 (48.0%)
	Other	711 (38.7%)
Previous total joint replacement	Hip	242 (13.2%)
	Knee	304 (16.5%)
Preoperative	None	58 (3.2%)
	Paracetamol	1061 (57.8%)
Medications taken for osteoarthritis	Non-steroidal anti-inflammatories	513 (27.9%)
	Opioids	372 (20.3%)
	Antidepressant/antiepileptics	34 (1.8%)
	Steroids	6 (0.3%)
Preoperative cardiac	Clopidogrel	64 (3.5%)
medications	Warfarin	68 (3.6%)
Preoperative hormone	replacement therapy	40 (2.2%)
ASA score (N = 1798)	1 or 2	1220 (67.9%)
	3 or 4	577 (32.1%)

The use of multiple medications for post-operative VTE prophylaxis was common (N = 738, 40.2%) (Table 3, Supplementary Table 1). The duration of prophylaxis was shorter than recommended for 18.8% (N = 612) THA and 14.7% (N = 558) TKA participants^7^.

**Table 3:** Site, surgeon, surgical and acute care characteristics.

Site & Surgeon	Description	Results N (%) or median (IQR)
Sites	Public	10 (54%)
	Private	9 (46%)
Surgeons		60 (30, 101)
Participants	Per surgeon	60 (30.0, 101.0)
	Per site	102.1 (49.3, 132.8)
Length of stay (days)		5 (4.0, 7.0)
Surgical and acute care		
Joint (all surgeries)	Hip	807 (43.9%)
	Knee	1031 (56.1%)
Bilateral THA/TKA	Hip	10 (0.5%)
	Knee	80 (4.4%)
Surgical duration (hours)	(N = 1837)	1.62 (1.2, 2.0)
Processes of care	Neuraxial anaesthesia (N = 1836)	1151 (62.7%)
	Routine doppler (N = 1810)	343 (19.0%)
	Cement fixation (N = 1837)	1172 (63.8%)
	Intra-articular drain (N = 1831)	801 (43.7%)
	Tourniquet (TKA) (N = 1032)	885 (48.2%)
	Blood transfusion (N = 1831)	316 (17.3%)
	Indwelling catheter	1433 (78.1%)
VTE Prophylaxis		
Mechanical	Calf compressors (N = 1810)	1410 (76.8%)
	Foot pumps (N = 1810)	277 (15.0%)
	Graduated compression stockings (GCS) (N = 1835)	1399 (76.2%)
	Used any SCD/GCS (N = 1835)	1837 (98.6%)
Duration (days)	SCD and GCS (N = 1835)	27 (15.0,38.0)
	Foot pumps only (N = 277)	4 (3.0, 5.0)
	Calf compressors only (N = 1410)	3 (2.0, 4.0)
First mobilised day 0 or 1		1376 (75.0%)
Chemical		
Number of drugs	0	7 (0.4%)
	1	1093 (59.5%)
	2	678 (36.9%)
	3	57 (3.1%)
	4	2 (0.1%)
Duration of chemoprophylaxis	22 (12,36) any duration	30.5 (13,48)
Preoperative use N (%)		
LMWH		1439 (78.3%)
Enoxaparin sodium	13 (0.7%)	1047 (57.0%)
Fragmin	0	392 (21.3%)
Aspirin (N = 1836)	458 (24.9%)	868 (47.3%)
First dose 100–300 mg	Not reported	847 (46.1%)
Rivaroxaban	13 (0.7%)	160 (8.7%)
Dabigatran etexilate	9 (0.5%)	11 (.6%)
Apixaban	2 (0.1%)	10 (0.5%)
Warfarin	66 (3.6%)	77 (4.2%)
Unfractionated heparin	4 (0.2%)	73 (4.0%)
Fondaparinux	0	0

### VTE non-compliance

Most participants were assessed as routine VTE risk (N = 1554, 82.9%), with 12.5% (N = 230) at high VTE risk and 2.9% (N = 53) at high bleeding risk, according to the AOA VTE CPG criteria (Table 4). The rate of compliance with patient-level risk-stratified compliant prophylaxis AOA VTE clinical guideline recommendations was 85.9% (N = 1570), including 1411 (76.8%) of the 1554 people considered at routine VTE risk who received routine VTE risk compliant prophylaxis and 168 (9.1%) of the 230 people considered at high VTE risk who received high VTE risk compliant VTE prophylaxis. No patient considered at high bleeding risk received care compliant for high bleeding risk prophylaxis. As overall compliance required people to receive care considered compliant with all three elements, only 14.0% (N = 258) of the whole cohort received care considered compliant with AOA recommended VTE prophylaxis. When considered by the assessed level of VTE and bleeding risk, nearly 30% of people at high VTE risk, and Forty-four people considered high VTE risk received 100–300 mg aspirin alone, which is only recommended for people at routine VTE risk. Nearly two thirds (N = 1137, 61.9%) of the cohort received prophylaxis for the minimum recommended duration of 21 days, and 42 (2.3%) people ceased prophylaxis to commence VTE treatment. Over two thirds 67.8% (N = 1245) were non-compliant with other general recommendations.

**Table 4:** Patient VTE risk and compliance with AOA VTE Prevention Guidelines (2018) (N = 1838).

Patient-level risk	N (%)
Criteria for high PE risk	
Major criteria (one or more):	174 (9.5%)
Minor criteria (three or more)^††^	96 (5.2%)
High VTE risk (≥ 1 major criteria or ≥ 3minor criteria)	230 (12.5%)
Routine VTE risk (did not meet high VTE risk criteria)	1554 (82.9%)
High risk of bleeding*** (known bleeding disorder)	53 (2.9%)
Prophylaxis received^^^	
(i) Sequential compression device	1683 (91.6)
(ii) Aspirin- 100–300 mg per day	836 (44.6%)
(iii) Potent anticoagulation (LMWH, Warfarin, DOAC)	1484 (79.1%)
Proportion of cohort who received prophylaxis compliant with routine and high VTE risk and high bleeding risk recommendations^^^	
(1) Compliant with routine recommended care (i. & ii &/or iii.)	1675 (91.2%)
(2) Compliant with high VTE risk recommendations (i and iii only, did not receive aspirin unless taking preoperatively)	1142 (62.2%)
(3) High bleeding risk VTE prophylaxis recommendations (i. only)	7 (0.4%)
Patients received recommended prophylaxis for the assessed risk level (Patient risk-stratified compliant prophylaxis)	
(1) People at routine VTE risk (N = 1554) who received routine VTE risk compliant prophylaxis	1411 (76.8%)
(2) High VTE risk (N = 230) who received high VTE risk compliant VTE prophylaxis	168 (9.1%)
(3) High bleeding risk (N = 54) who received high bleeding risk compliant VTE prophylaxis	0 (0%)
Elements of compliance	
A. Received risk-stratified recommended prophylaxis (1 + 2 + 3)	1579 (85.9%)
Recommended duration	
Three to six weeks (recommended)	1035 (56.3%)
(i) At least 21 days (considered compliant)	1137 (61.9%)
(ii) Ceased prophylaxis to start VTE treatment (compliant)	42 (2.3%)
B. Compliant with recommended duration (i. or ii)^¥^	1169 (63.6%)
Other general recommendations	
(i) Received neuraxial anaesthesia (N = 1837) (1179 compliant)	1151 (62.7%)
(ii) Neuraxial anaesthesia contraindicated (on warfarin)	68 (3.6%)
(iii) Bleeding mitigation (received tranexamic acid)	1115 (59.5%)
(iv) Ceased preoperative warfarin within 5 days of surgery^‡^	37 (2.0%)
(v) Ceased preoperative clopidogrel within 5 days of surgery^‡^	45 (2.5%)
(vi) Early mobilisation (walked day 0 or day 1)	1376 (75.0%)
C. Compliant with all relevant general recommendations (i. to vi.)	592 (32.2%)
Overall AOA compliance (compliant with A and B and C)	258 (14.0%)

***30 people at high bleeding risk also met criteria for high VTE risk and were considered at high bleeding risk.

^††^12 people met criteria for both minor and major high-risk factors for PE.

^^^Regardless of patient level risk, categories not exclusive.

^¥^10 people had at least 21 days prophylaxis duration and eased VTE prophylaxis to commence treatment.

^‡^Only considered if taking preoperative warfarin or clopidogrel.

### Participant outcomes: surgical complications up to 90 days

The incidence of symptomatic VTE was 2.6% (N = 48), with nearly two-thirds of the cases experiencing DVT alone (Table 5). Bleeding complications requiring readmission or reoperation were rare (17, 0.92%) (Table 5, Supplementary Table 2). Four participants died from surgical complications, and seven died from medical causes.

**Table 5:** Prevalence of VTE events up to 90-days.

Type and timeframe	Event number		
Type	1st	2nd	Totals
PE and DVT	2 (0.1%)	0	2
PE only	15 (1.0%)	2	17
Symptomatic DVT	31 (1.7%)	2	33
Totals	48 (2.6%)	4 (0.2%)	52 (2.8%)
Time period			
Acute admission	18 (1.0%)	0	18 (1.0%)
Acute discharge to 35 days	24 (1.3%)	3 (0.2%)	27 (1.5%)
36 to 90 days	6 (0.3%)	1 (0.1%)	8 (0.4%)
Totals	48 (2.6%)	4 (0.2%)	53 (2.9%)

### Association between VTE prophylaxis non-compliance and VTE outcomes

In unadjusted analyses, non-compliance with AOA VTE prevention clinical guidelines was not significantly associated with lower symptomatic 90-day VTE (Supplementary Table 3).

In adjusted analyses, overall non-compliance with AOA VTE prevention clinical guidelines recommendations was not significantly associated with the risk of symptomatic VTE at 90 days (AOR = 0.93, 95%CI = 0.4 to 1.3, p = 0.86)^5^ (Table 6, Supplementary Table 4). There was no collinearity between variables and no significant interaction terms. The effect estimates for VTE prevention clinical guidelines non-compliance remained nonsignificant when people with high bleeding risk were excluded (AOR = 0.94, 95%CI = 0.44 to 2.34, *p* = 0.89), when people with a history of VTE were excluded (AOR = 1.17, 95%CI = 0.50 to 3.46, *p* = 0.74), when people who received a routine DUS were excluded (AOR = 0.85, 95%CI = 0.37 to 2.31, *p* = 0.73), and when people with THA or TKA were analysed separately (THA: AOR = 0.44, 95%CI = 0.12 to 2.06, *p* = 0.24; TKA: AOR = 1.39, 95%CI = 0.54 to 4.70, *p* = 0.54).

**Table 6:** Final regression model for association between AOA VTE prevention clinical guideline non-compliance and symptomatic 90-day VTE outcomes.

Variables	AOR (95% CI)	*p*-value
Overall AOA VTE CPG non-compliance	0.93 (0.4–1.3)	0.86
TKA	2.1 (0.9–1.0)	0.09
History of previous VTE	0.9 (0.8–4.1)	0.1
Received routine DUS	1.8 (0.9–3.6)	0.08
Cement fixation	1.9 (0.8–3.4)	0.17

## Discussion

Non-compliance with the AOA VTE prevention clinical guidelines was not significantly associated with the risk of VTE. We reported higher levels of non-compliant prophylaxis for people assessed as being at high VTE risk (27.0%) and high bleeding risk (100%), compared to those at routine risk (9.2%) (using AOA criteria). All people considered at high bleeding risk received chemical prophylaxis, despite this not being recommended for this group.

Adherence to AOA VTE prevention clinical guideline recommendations does not appear to reduce the risk of VTE after THA and TKA. These findings are consistent with the US Surgical Care Improvement Project (SCIP), which reported a higher rate of PE in those who received care compliant with the VTE process measures (doctor ordered VTE prophylaxis, and the patient received VTE prophylaxis at the right time)^14,15^. This program uses process of care elements that are also very broad and may mask any differential outcome associated with different types of chemoprophylaxis^16^. Compliance with AOA recommended VTE chemical prophylaxis is flexible and surgeons are given discretion to use aspirin (100–300 mg), low molecular weight heparins or DOACs for people at routine VTE risk, although in this study aspirin was also used for people assessed as high VTE risk^5^. While it was not within the scope of this study, further research should explore why some surgeons used this flexibility with discretion to accommodate individual patient needs, while others prescribed noncompliant prophylaxis. These results may inform future revision of the AOA VTRE Prevention Guidelines.

While nonsignificant, we reported a trend towards a reduction in risk associated with non-compliant VTE prophylaxis. This contrasts with our previous analysis demonstrating noncompliance with the NHMRC VTE guideline was significantly associated with increased risk of VTE^17^. The main difference in the recommended prophylaxis is that aspirin is considered compliant for AOA VTE prevention clinical guidelines, but was not considered compliant for NHMRC VTE prevention clinical guideline^5,7^. The use of aspirin remains controversial although it is now recommended in several VTE clinical guidelines when used as part of a comprehensive VTE prophylaxis program. VTE, SSI and bleeding outcomes for aspirin prophylaxis have been shown to be no different to potent anticoagulants^18–20^. However other studies have reported aspirin is associated with an increased incidence of DVT and wound complications, but a lower risk of bleeding^21–23^. There has been a call for high quality RCT to confirm the efficacy and safety profile of aspirin, and several are now underway^1,18,24,25^.

This study has several limitations. The AOA guidelines target PE rather than DVT^26^; we included symptomatic DVT as both are associated with higher costs, lower patient satisfaction and are a target for Australian hospital accreditation^27,28^. The low event rate for PE prevented sensitivity analyses for PE alone. Calculation of patient risk was constrained by the available data; alternative criteria for compliance may yield different results. We may not have accounted for all the variation in prophylactic regimens. We did not assess the quality of the AOA VTE prevention clinical guidelines as the lack of peer-reviewed evidence regarding the methods or impact of the AOA VTE clinical guidelines precluded formal assessment. Research has demonstrated variable quality and issues with implementing other VTE clinical guidelines^9,29^, even though the patient and system benefits of increasing compliance^14,30^ can only be realised if the guidelines are of the highest quality^8,9^. Finally, our sample included a higher rate of THA and surgery performed in public hospitals than national registry data and the findings may not be generalisable to other populations^5^.

A key strength of this study lies in the comprehensive prospective clinical data to assess risk stratified VTE prophylaxis and compliance with the AOA VTE prevention clinical guidelines. Patient appropriate variations were considered in assessing compliance^31,32^. It is unclear whether the use of aspirin was associated with the increased VTE events we saw with people who experienced VTE while taking prophylaxis. Other potential factors are the AOA VTE prevention clinical guidelines recommendations are too broad to impact risk or a consequence of the varying recommendations between different guidelines or other factors^28,33^. Further research is recommended to explore factors influencing individualised risk assessment and prophylaxis, and the association with economic and patient reported outcomes^27^.

## Conclusion

The aim of clinical guidelines is to help clinicians apply evidence from clinical trials into real-world practice and improve patient outcomes^31^. In contrast to this aim, we found non-compliance with AOA VTE prevention clinical guidelines was not associated with the risk of symptomatic 90-day VTE after THA/TKA, suggesting that these guidelines are not effective at reducing VTE events. The lack of data regarding the methodological quality and evidence underpinning the AOA VTE prevention clinical guidelines is concerning^8,9^. VTE is associated with high cost and burden for patients and the health system^34^. Definitive studies are needed to validate individualised VTE risk assessments and confirm effective personalised VTE prophylaxis strategies, particularly for those considered high risk, to improve patient outcomes and the value of arthroplasty surgery.

### Supplementary Information

Supplementary Information.

## Data Availability

A deidentified version of the data set and the full R code for all analyses are available in the Australian Research Data Commons (10.26190/c46r-ne05).

## Abbreviations

AIC: Akaike information criterion
AOR: Adjusted odds ratio
ASA: American Society of Anesthesiology
BIC: Bayesian information criterion
BMI: Body mass index
DVT: Deep vein thrombosis
GCS: Graduated compression stockings
GORD: Gastro-intestinal reflux disorder
ICD: Intermittent compression devices
ICP: Intermittent compression pumps (type of ICD)
IQR: Inter-quartile range
LOS: Length of stay
LTFU: Lost to follow up
LMWH: Low molecular weight heparins
MICE: Multivariate imputation by chained equations
NHMRC: National Health and Medical Research Council
NSAID: Non-steroidal anti-inflammatories
PE: Pulmonary embolism
SCD: Sequential compressive devices
SD: Standard deviation
SE: Standard error
THA: Total hip arthroplasty
TKA: Total knee arthroplasty
VTE: Venous-thromboembolism
