# Supplementary material for: Compliance with Australian Orthopaedic Association guidelines does not reduce the risk of venous thromboembolism after total hip and knee arthroplasty
Source: Sci Rep. 2024 Mar 12;14:5955. doi: 10.1038/s41598-024-54916-x (PMC10928067; doi:10.1038/s41598-024-54916-x)
Supplement: Supplementary file 1 — Supplementary Information. [file 41598_2024_54916_MOESM1_ESM.docx]

# Compliance with Australian Orthopaedic Association guidelines does not reduce the risk of venous thromboembolism after total hip and knee joint arthroplasty: an observational study

**Authors’ information**

Helen Badge^1,2,3,4^¶*, Tim Churches^2,3^¶, Justine M Naylor^1,2,3^¶, Wei Xuan^2,3^¶, Elizabeth Armstrong⁵, Leeanne Gray^6^, John Fletcher⁷^,^⁸, Iain Gosbell³^,^⁹, Chung-Wei Christine Lin^10^ & Ian A Harris^1,2, 3,6^¶

**Author details**

^1^ Whitlam Orthopaedic Research Centre, 1 Campbell Street, Liverpool, 2071, Australia

^2^ South Western Sydney Clinical School, UNSW, 1 Elizabeth Street, Liverpool, 2071, Australia

^3^ Ingham Institute for Applied Medical Research, 1 Campbell Street, Liverpool, 2071, Australia

^4^ Australian Catholic University, 8-20 Napier Street, North Sydney, 2060, Australia

⁵ School of Public Health, The University of New South Wales, High St Kensington, NSW 2052, Australia

^6^ South Western Sydney Local Health District, 1 Elizabeth Street, Liverpool, 2071, Australia

⁷ University of Sydney, Fisher Road, Camperdown, NSW, 2006, Australia

⁸ Westmead Hospital, Cnr Hawkesbury Road and Darcy Road, Westmead, NSW, 2145, Australia

⁹ Western Sydney University, Campbelltown, NSW, 2560, Australia

^10^ Sydney School of Population Health, The University of Sydney, Edward Ford Building (A27) Fisher Road, Camperdown, NSW, 2006, Australia

*Corresponding author: [helen.badgehawke@gmail.com](mailto:helen.badge@acu.edu.au)

HB is supported by an Australian Government Research Training Program Scholarship.

**SUPPLEMENTARY FILE 1**

**Supplementary Table 1: Additional description of prophylaxis up to 90 days**

| **VTE prophylaxis** | **Description** | **N (%) or**  **Median (IQR)** |
| --- | --- | --- |
| Clexane (Enoxaparin)  (N=1047/1837)  Median (IQR) | Received  First dose  20mg  40mg  60-80mg  100-200mg  Dose changed after 1^st^ dose  Day commenced  Duration (days)  Days break in prophylaxis  Number with disrupted prophylaxis | 1047 (57.0%)  51 (2.8%)  980 (53.4%)  10 (0.5%)  4 (0.4%)  42 (2.3%)  0 (0,1)  11 (5.0, 20.0)  2 (1,3)  63 (3.4%) |
| Fragmin (Dalteparin)  Median (IQR) | Received  First dose (International Unit, IU)  2500 IU  5000 IU  10000 IU  Dose changed after 1^st^ dose  Day commenced  Duration (days)  Days break in prophylaxis  Number with disrupted prophylaxis | 392 (21.3%)  124 (6.7%)  265 (14.4%)  1 (0.1%)  7 (0.4%)  0 (0,1)  10 (5.0, 19.0)  2 (1,2)  22 (1.2%) |
| Aspirin (acetylsalicylic acid)  Median (IQR) | Received  First dose  <100mg  100mg  150mg  200mg / 250mg  300mg  >300 mg  Dose changed after 1^st^ dose  Day commenced  Duration (days)  Days break in prophylaxis  Number with disrupted prophylaxis | 869 (47.3%)  13 (0.7%)  532 (28.9%)  29 (1.6%)  3 (0.1%)  273 (14.9%)  1 (0.1%)  23 (1.3%)  2 (1,7)  42 (34,90)  2 (2,5)  29 (1.6%) |
| Rivaroxaban  Median (IQR) | Received  First dose  10mg  15mg  20mg  Dose changed after 1^st^ dose  Day commenced  Duration (days)  Days break in prophylaxis  Number with disrupted prophylaxis | 160 (8.7%)  129 (7.0%)  5 (0.3%)  13 (0.7%)  20 (1.1%)  6 (4,8)  19.5 (13,30)  0.5 (0.2,0.8)  2 (0.1%) |
| Dabigatran etexilate  Median (IQR) | Received  First dose  110mg / 125mg  150mg  220mg / 300mg  Dose changed after 1^st^ dose  Day commenced  Duration (days)  Days break in prophylaxis  Number with disrupted prophylaxis | 11 (.6%)  3 (0.2%)  4 (0.2%)  4 (0.2%)  1 (0.1%)  3 (1.0, 6.5)  90 (90,90)  NA  0 |
| Warfarin (Vitamin K antagonist)  Median (IQR) | Received (doses variable)  Day commenced  Duration (days)  Days break in prophylaxis  Number with disrupted prophylaxis  Recommenced preoperative medication | 77 (4.2%)  2 (1,6)  90 (90,90)  3 (2,4)  3 (0.2%)  69 (3.8%) |
| Unfractionated heparin  Median (IQR) | Received  First dose (N=71)  1500U /1600U  5000U  10000U  variable  Dose changed after 1^st^ dose  Day commenced  Duration (days)  Days break in prophylaxis  Number with disrupted prophylaxis | 74 (3.9%)  42 (2.5%)  17 (0.8%)  9 (0.8%)  1 (0.1%)  3 (0.2%)  0 (0,1)  1 (1,6)  0 (0,1)  1 (0.1%) |
| Apixaban  Median (IQR) | Received  First dose  2.5mg  5mg  10mg  Dose changed after 1^st^ dose  Day commenced  Duration (days)  Days break in prophylaxis  Number with disrupted prophylaxis | 10 (0.5%)  6 (0.3%)  2 (0.1%)  2 (0.1%)  0  8 (7,10)  15 (15,90)  NA  0 (0%) |
| Sequential compression devices: ICP | Received  Day commenced  Duration (days) | 1410 (76.8%)  0 (0,0)  3 (2,4) |
| Sequential compression devices: Foot pumps | Received  Day commenced  Duration (days) | 277 (15.0%)  0 (0,0)  4 (3,5) |
| Graduated compression stockings | Received  Day commenced  Duration (days) | 1400 (76.2%)  0 (0,0)  23 (14,42) |

Supplementary Table 2: Prevalence of bleeding complications up to 90 days post-surgery

| **Highest type of bleeding complications ^1^** | **Joint related bleeding** †† | **Non-joint related bleeding** |
| --- | --- | --- |
| Type 1 (no healthcare action) | 9 (0.5%) | 1 (0.1%) |
| Type 2 (overt bleed, healthcare provided) | 35 (1.9%) | 8 (0.4%) |
| Type 3 (major intervention, ICU admission / readmission / reoperation) | 7 (0.4%) | 10 (0.5%) |
| Type 5 (Fatal) | 0 | 0 |
| TOTALS | 51 (2.8%) | 19 (1.0%) |
| **Time period of bleeding complications** | **Joint related bleeding** | **Non-joint related bleeding** |
| During acute admission | 36 (1.9%) | 8 (0.4%) |
| Between acute discharge and 35 days | 14 (0.8%) | 11 (0.6%) |
| Between 36-90 days | 1 | N/A |
| TOTALS | 51 (2.8%) | 20* (1.1%) |

†† Included one person who experienced two bleeding events: one during acute admission and another between acute discharge and 35 days (type 1 and 3)

Supplementary Table 3: Unadjusted associations between compliance with AOA VTE prevention clinical guidelines and symptomatic 90-day VTE

| **Variables** | **Total** | **No VTE,**  **N (%)**  **(N=1789)** | **Symptomatic 90-day VTE, N (%) (N=48)** | **p-value** |
| --- | --- | --- | --- | --- |
| **AOA noncompliance** |  |  |  |  |
| 1. Risk-stratified recommended prophylaxis   Non-compliant  Compliant | 258 (14.0%)  1579 (86.0%) | 252 (97.7%)  1537 (97.3%) | 6 (2.3%)  42 (2.7%) | 1.00 |
| 1. Recommended duration   Non-compliant  Compliant | 668 (36.4%)  1169 (63.6%) | 665 (99.6%)  1124 (96.2%) | 3 (0.4%)  45 (3.8%) | <0.0001 |
| 1. Other general recommendations   Non-compliant  Compliant | 1245 (67.8%)  592 (32.2%) | 1207 (96.9%) 582 (98.3%) | 38 (3.1%)  10 (1.7%) | 0.12 |
| AOA recommended prophylaxis  (Compliant with i., ii & iii)  Non-compliant  Compliant | 1570 (85.5%)  267 (14.5%) | 1529 (97.4%)  260 (97.4%) | 41 (2.6%)  41 (2.6%) | 1.00 |
| **Other factors** |  |  |  |  |
| VTE risk* (N=1784)  Routine  High | 1554 (87.1%)  230 (12.91%) | 1515 (97.5%)  222 (96.5%) | 39 (2.5%)  8 (3.5%) | 0.40 |
| Joint  THA  TKA | 807 (43.9%)  1030 (56.1%) | 797 (98.8%)  991 (96.3%) | 10 (1.2%)  38 (3.7%) | 0.001 |
| Routine DUS (N=1810)  No  Yes | 1467 (81.0%)  343 (19.0%) | 1433 (97.7%)  329 (95.9%) | 34 (2.3%)  14 (4.1%) | 0.09 |
| History of previous VTE (N=1835)  No  Yes | 1690 (92.1%)  145 (7.9%) | 1649 (97.6%)  138 (95.2%) | 41 (2.4%)  7 (4.8%) | 1.00 |
| Cement fixation  No  Yes | 664 (36.2%)  1172 (63.8%) | 656 (98.8%)  1132 (96.6%) | 8 (1.2%)  40 (3.4%) | 0.004 |
| Neuraxial anaesthesia (N=1836)  No  Yes | 657 (35.8%)  1179 (64.2%) | 645 (98.2%)  1143 (96.9%) | 12 (1.8%)  36 (3.1%) | 0.13 |
| Sequential compression device  No  Yes | 154 (8.4%)  1683 (91.6%) | 151 (98.1%)  1638 (97.3%) | 3 (1.9%)  45(2.7%) | NA** |
| Aspirin- 100-300mg per day  No  Yes | 1003 (54.6%)  834 (45.4%) | 972 (96.9%)  817 (98.0%) | 31 (3.1%)  17 (2.0%) | 0.18 |
| Potent anticoagulation (LMWH, Warfarin, DOAC)  No  Yes | 355 (19.3%)  1482 (80.7%) | 349 (98.3%)  1440 (97.2%) | 6 (1.7%)  42 (2.8%) | 0.3 |
| Early mobilisation (N=1834)  No  Yes | 458 (25.0%)  1376 (75.0%) | 437 (95.4%)  1349 (98.0%) | 21 (4.6%)  27 (2.0%) | 0.004 |
| Tranexamic acid (N=1833)  No  Yes | 718 (39.2%)  1115 (60.8%) | 700 (97.5%)  1085 (97.3%) | 18 (2.5%)  30 (2.7%) | 0.9 |
| **Continuous variables** |  | Mean | Mean | Mean |
| Age (years) |  | 67.2 | 68.8 | 0.22 |
| BMI |  | 30.7 | 33.3 | 0.05 |
| Surgical duration (hours) |  | 1.6 | 1.8 | 0.22 |

*Excluding 53 people at high bleeding risk **Chi square not performed due to low cell count

**Supplementary Table 4: Eligible factors included in the initial adjusted model**

| Type of factor | Description |
| --- | --- |
| Compliance factors | - AOA non-compliance |
| Patient factors | - Age - Sex - ASA score - Presence of comorbid conditions (previous VTE, previous THA, heart disease) - Current smoker |
| Surgical and care factors | - THA/TKA - Public or private hospital - Cement fixation - Surgical drain - Routine doppler performed - Surgical duration |

1 Mehran, R. *et al.* Standardized bleeding definitions for cardiovascular clinical trials. *Circulation* **123**, 2736-2747, doi:10.1161/circulationaha.110.009449 (2011).
